# Impact of Platelets to Lymphocytes Ratio and Lymphocytes during Radical Concurrent Radiotherapy and Chemotherapy on Patients with Nonmetastatic Esophageal Squamous Cell Carcinoma

**DOI:** 10.1155/2022/3412349

**Published:** 2022-04-29

**Authors:** Yaotian Zhang, Ning Han, Xue Zeng, Chaonan Sun, Shichen Sun, Xinchi Ma, Yanyu Zhang, Zhuang Liu, Zilan Qin, Hong Guo, Yubing Li, Na Zhang

**Affiliations:** Department of Radiation Oncology, Cancer Hospital of China Medical University, Liaoning Hospital and Institute, Shenyang Liaoning 110042, China

## Abstract

**Purpose:**

This study examined the importance of hematological parameters as prognostic markers for people with esophageal cancer receiving radical concurrent chemoradiation.

**Methods:**

106 patients with esophageal cancer are included in this study. Cox regression analysis, Kaplan-Meier method, and chi-square test were used to analyze our data.

**Results:**

The median follow-up time for patients was 15.5 months (3-55). Univariate and multivariate analyses showed that age, the change of platelet-to-lymphocyte ratio (*Δ*PLR), and the change rate of circulating lymphocyte count (*Δ*CLC%) were independent influencing factors of OS and DFS. The patients were grouped according to the median of *Δ*PLR and *Δ*CLC%, and analysis showed that a higher *Δ*PLR and a higher *Δ*CLC% was related to poor OS and DFS (*P* < 0.001, *P* < 0.001 and *P* < 0.001, *P* < 0.001). By subgroup analysis, the OS of T1-4N1-2 were better in the low *Δ*PLR group than the high one (*P* = 0.03, *P* < 0.001, *P* = 0.001, *P* < 0.001, and *P* = 0.008). DFS of T3-4N1-2 in the low *Δ*PLR group were better than the high one (*P* < 0.001, *P* = 0.016 and *P* < 0.001, *P* = 0.022). For patients with T1-4N0-2, the OS in the low *Δ*CLC% group were better than in the high *Δ*CLC% group (*P* = 0.01, *P* < 0.001, *P* < 0.002, *P* = 0.012, *P* < 0.001, and *P* = 0.024). For T1-4N1-2, the DFS were better in the low *Δ*CLC% group than others (*P* = 0.042, *P* < 0.001, *P* < 0.001, *P* < 0.001, and *P* = 0.006).

**Conclusion:**

*Δ*PLR and *Δ*CLC% are independent factors of OS and DFS, and a lower *Δ*PLR and *Δ*CLC% are associated with a better OS and DFS. And T3-4N1-2 patients in the low *Δ*PLR group and low *Δ*CLC% group have greater survival benefit.

## 1. Introduction

China is one of the countries with an high risk in esophageal carcinoma, and esophageal squamous cell carcinoma (ESCC) accounts for 90% of the national cases [1]. Recently, there are many factors influence clinical outcomes. Among them, TNM stage is more influential and more acceptable [2]. However, the value of TNM stage is unclear. Patients with same TNM stage have various outcomes. Therefore, the new biomarker should be found to predict more precise outcomes for people with ESCC.

Above 70% of esophageal carcinoma patients have malnutrition [3], malnutrition is associated with poor survival, and it increases complications [4]. Studies shown that malnutrition can increase blood system toxicity [5]. Many studies have shown that tumor-related factors including inflammatory factors and nutritional status are also related to the prognosis of tumor patients [6–20]. Evidence has shown that monocytes/lymphocytes ratio (MLR) maybe an effective prognostic indicator of tumors [21, 22]. The study by Xiao et al. [23] also found that the low NLR before neoadjuvant chemoradiation for esophageal cancer was significantly associated with postoperative pCR, and the lower PLR after neoadjuvant chemoradiation was also associated with pCR. In addition to NLR, treatment-related lymphopenia is a powerful factor in the poor prognosis of esophageal [14, 15, 24]. Severe lymphopenia during neoadjuvant concurrent radiotherapy and chemotherapy is associated with adverse pathological reactions and recurrence of cancer [25, 26]. These markers are cheap and easy to obtain, so they are expected to act as clinical prognostic factors of cancer.

Now, the relationship between changes of inflammatory factors during CCRT of esophageal cancer and prognosis has rarely been shown. What our study wants to research is to determine the influence of inflammatory factors on the prognosis of patients with esophageal cancer undergoing radical concurrent radiotherapy and chemotherapy.

## 2. Materials and Methods

### 2.1. Patients

**Figure 1:**
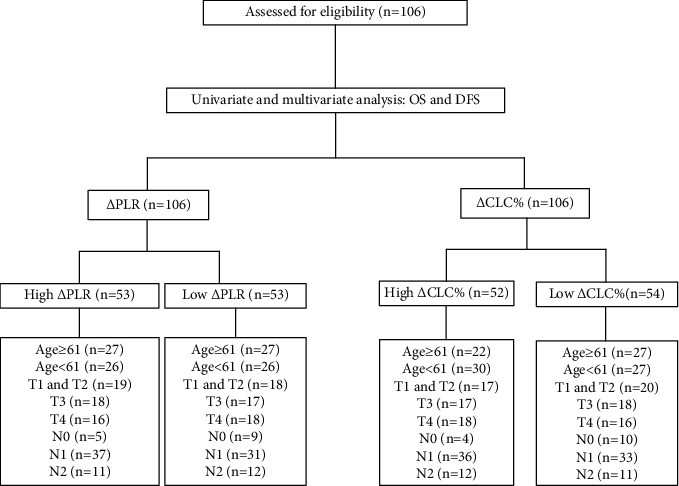
Overall study flow chart. Abbreviations: OS: overall survival; DFS: disease-free survival; PLR: platelet-to-lymphocyte ratio; CLC: circulating lymphocyte count.

106 newly diagnosed patients with ESCC, who received radical concurrent radiotherapy and chemotherapy (CCT) between January 2016 and December 2017, were included in this study (Figure 1). The 8^th^ edition of AJCC system was used to stage the patient [2]. Patients should have at least two routine blood tests, one should be within 2 weeks before radiotherapy, and the other should be within 1 month after radiotherapy. If patient has undergone surgery and has concomitant diseases that may affect the count of white blood cells, neutrophils, lymphocytes, platelets, etc., including inflammation, autoimmune diseases, history of blood transfusion, liver cirrhosis, spleen disease, and severe hypertension will be excluded. The ethics committee of Liaoning Cancer Hospital permitted this study. We have got the consent.

### 2.2. Radiation

IMRT with 6 megavoltage (MV) photons was given to total patients. The prescribed doses were defined as follows: 60-64 Gy for CTV. Each dose was divided into 30-32 fractions.

### 2.3. CCT

CCT was cisplatin (75 mg/m^2^, days 1-3) and fluorouracil (750-1000 mg/m^2^, CIV24h, d1-4) which was given to all patients. All patients received two cycles of chemotherapy during radiotherapy.

### 2.4. Inflammatory Factors

Eight parameters are the inflammatory factors, namely, changes during CCRT in the neutrophil-to-lymphocyte ratio (*Δ*NLR), the PLR (*Δ*PLR), the platelet (*Δ*PLT), the circulating lymphocyte count (*Δ*CLC), change rates during CCRT in the NLR (*Δ*NLR%), the PLR (*Δ*PLR%), the platelet (*Δ*PLT%), the circulating platelet count (*Δ*CPC%), and the circulating lymphocyte count (*Δ*CLC%).

NLR1, PLT1, PLR1, and CLC1 are the count before radiotherapy. NLR2, PLT2, PLR2, and CLC2 are the count after radiotherapy (NLR2): ΔNLR = NLR1 − NLR2, ΔNLR% = (NLR1 − NLR2)/NLR1, ΔPLR = PLR1 − PLR2, ΔPLR% = (PLR1 − PLR2)/PLT1, ΔCLC = CLC1 − CLC2, and ΔCLC% = (CLC1 − CLC2)/CLC1.

### 2.5. Statistics

The Cox proportional hazard regression model was used to analyze the prognostic factors affecting disease-free survival (DFS) and overall survival (OS). Variables with *P* < 0.05 were included in a multivariate analysis. Subgroup analyses were performed using Chi-square test. The rates of DFS and OS were estimated with the Kaplan–Meier method and compared with the log-rank test. All data were analyzed using SPSS 22.0 software package (IBM Corporation, Armonk, NY, USA).

## 3. Result and Discussion

### 3.1. Patient Characteristics

106 patients with ESCC were included in the study, including 102 males (96.2%) and 4 females (3.8%), and received radical concurrent radiotherapy and chemotherapy (CCRT) (Table 1).

### 3.2. Follow-Up and Hematological Parameters

The median OS of the patients was 15.5 months (3-55 months), the median DFS was 10 months (1-55 months) in our study. By analyzing the relationship between hematological parameters and OS and DFS, we found that there was a significant correlation between *Δ*PLR and *Δ*CLC% and OS and DFS. After Cox regression univariate analysis, *Δ*PLR, *Δ*PLR%, *Δ*NLR, *Δ*NLR%, *Δ*CLC, and *Δ*CLC% were the independent factors of OS and DFS, *Δ*PLR, *Δ*PLR%, and age are the independent factors of OS (Table 2).

All factors such as age, *Δ*PLR, *Δ*PLR%, *Δ*NLR, *Δ*NLR%, *Δ*CLC, and *Δ*CLC% are used into multivariate analysis. Age and *Δ*PLR were the independent factors of OS (*P* = 0.028, HR = 0.961; *P* = 0.030, HR = 0.998), and *Δ*CLC% was an independent influence factor of DFS (*P* = 0.024, HR = 1.044) (Table 3).

**Figure 2:**
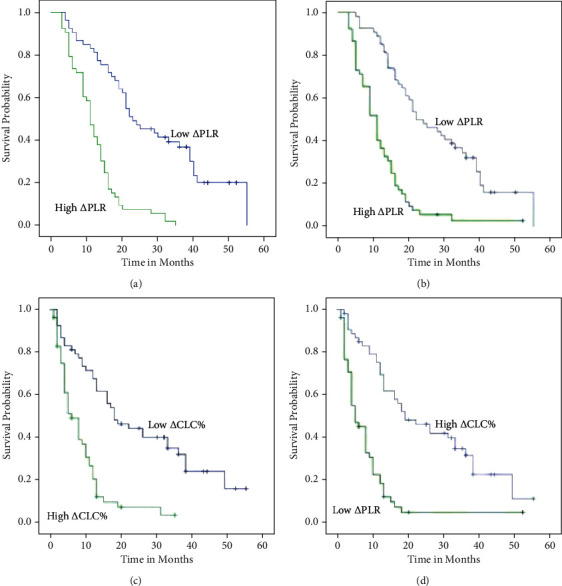
Kaplan-Meier plots of OS (a) and PFS (b) stratified by *Δ*PLR and OS (c) and PFS (d) stratified by *Δ*CLC%.

The median was used as the cut-off value for grouping. The high *Δ*PLR group refer to the absolute value of ΔPLR ≥ 290.72, and the low *Δ*PLR group was the absolute value of ΔPLR < 290.72. Comparing the clinical characteristics (Table 4) and OS and DFS between the two groups, the OS in the low *Δ*PLR group was better than the high *Δ*PLR group (95% CI: 12.838-17.162, *P* < 0.001) (Figure 2(a)), and the DFS was also better than the high *Δ*PLR group (95% CI: 8.340, 13.660, *P* < 0.001) (Figure 2(b))). the radiation pneumonitis of the low *Δ*PLR group was better than that of the high *Δ*PLR group (*P* = 0.027), but there was no significant difference in gender, age, tumor TNM stage, smoking history, drinking history, and radiation esophagitis The high *Δ*CLC% group was defined as ΔCLC% ≥ 75.51, and the low *Δ*CLC% group was defined as ΔCLC% < 75.51.

The basic clinical characteristics (Table 5) and OS and DFS of the two groups were compared. The basic clinical characteristics in two groups have no difference. The OS with the low *Δ*CLC% group was better than the high one (95% CI: 12.838, 17.162, *P* < 0.001) (Figure 2(c)), and DFS was also significantly better than high group (95% CI: 8.340, 13.660, <0.001) (Figure 2(d)).

### 3.3. Subgroup Analysis

We make patients into different subgroups by the T stage (T1-2, T3, and T4), N stage (N0-2 and N3), and age (≥61 and <61). TNM staging is closely related to the prognosis, and age in this study was an independent prognostic factor of OS.

**Figure 3:**
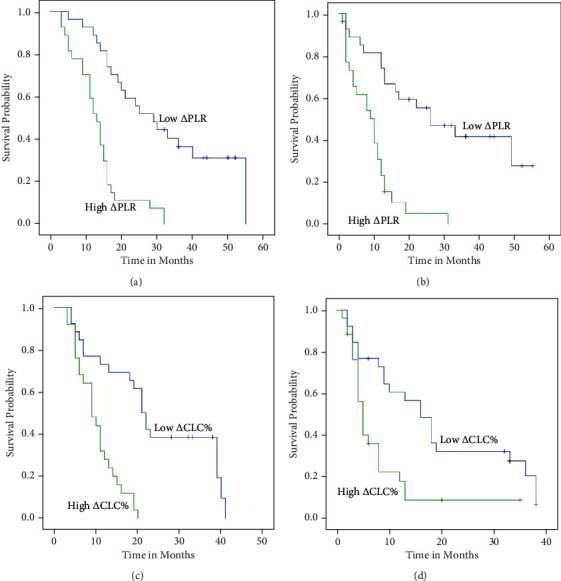
Kaplan-Meier plots of OS (a) and PFS (b) among patients whose age ≥ 61 stratified by *Δ*PLR and OS (c) and PFS (d) among patients whose age < 61 stratified by *Δ*PLR.

For age, the OS and DFS were batter in the low group than those in the high group (Figure 3).

**Figure 4:**
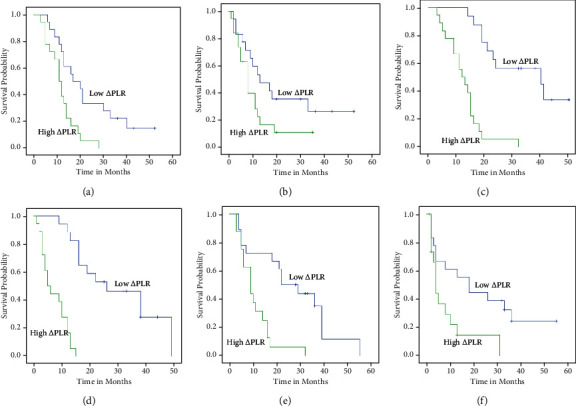
Kaplan-Meier plots of OS (a) and PFS (b) among patients with T1-2, OS (c) and PFS (d) among patients with T3, and OS (e) and OS (f) with T4 stratified by *Δ*PLR.

**Figure 5:**
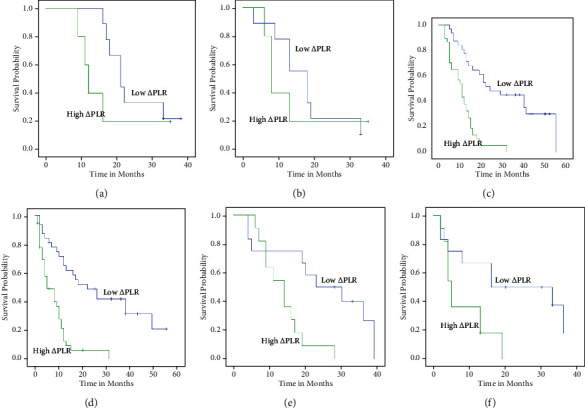
Kaplan-Meier plots of OS (a) and PFS (b) among patients with N0, OS (c) and PFS (d) among patients with N1, and OS (e) and OS (f) with N2 stratified by *Δ*PLR.

For patients with T1-4, the OS in the low *Δ*PLR group were better than the high *Δ*PLR group (*P* = 0.03, *P* < 0.001, and *P* = 0.001) (Figures 4(a), 4(c), and 4(e)). For patients with N2-3, the OS were better than the high *Δ*PLR group, too (*P* < 0.001 and *P* = 0.008) (Figures 5(a), 5(c), and 5(e)). For patients with T3-4N1-2, the DFS in the low *Δ*PLR group were better than in the high *Δ*PLR group (*P* < 0.001, *P* = 0.016 and *P* < 0.001, *P* = 0.022) (Figures 4(b), 4(d), 4(f), 5(b), 5(d), and 5(f); Table 6).

**Figure 6:**
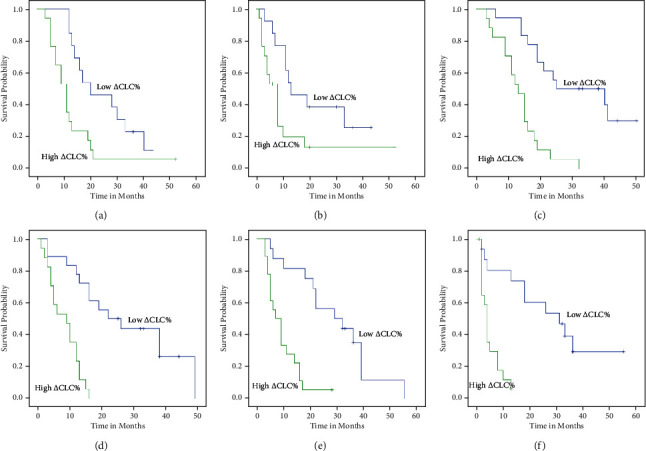
Kaplan-Meier plots of OS (a) and PFS (b) among patients with T1-2, OS (c) and PFS (d) among patients with T3, and OS (e) and OS (f) with T4 stratified by *Δ*CLC%.

**Figure 7:**
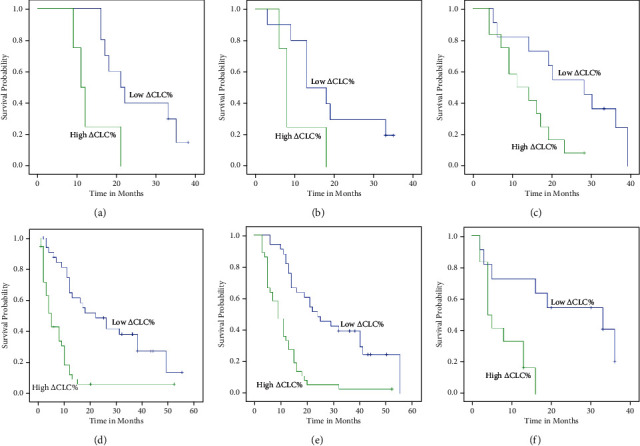
Kaplan-Meier plots of OS (a) and PFS (b) among patients with N0, OS (c) and PFS (d) among patients with N1, and OS (e) and OS (f) with N2 stratified by *Δ*CLC%.

For patients with T1-4N0-2, the OS in the low *Δ*CLC% group were better than the high *Δ*CLC% group (*P* = 0.01, *P* < 0.001, *P* < 0.002, *P* = 0.012, *P* < 0.001, and *P* = 0.024) (Figures 6(a), 6(c), 6(e), 7(a), 7(c), and 7(e)). For patients with T1-4N1-2, the DFS were better in the low *Δ*CLC% group than the high *Δ*CLC% group (*P* = 0.042, *P* < 0.001, *P* < 0.001, *P* < 0.001, and *P* = 0.006) (Figures 6(b), 6(d), 6(f), 7(d), and 7(f); Table 7).

## 4. Discussion

Radiotherapy is the indispensable treatment methods of esophageal cancer [27]. In our study, we studied 106 patients with ESCC who received radical concurrent radiotherapy and chemotherapy. *Δ*PLR and *Δ*CLC% during treatment are related to survival. During radiotherapy, the more *Δ*PLR and *Δ*CLC% fluctuate, the poorer patients survive. We included the patient's age, *Δ*PLR, *Δ*PLR%, *Δ*NLR, *Δ*NLR%, *Δ*CLC, *Δ*CLC%, and other factors into the Cox analysis. *Δ*PLR and *Δ*CLC% are, respectively, related to OS and DFS. Grouped by median, the prognosis of the low *Δ*PLR group and the low *Δ*CLC% group were better, and the difference between these two groups was obvious. Bone marrow suppression was a common side effect of concurrent radiotherapy and chemotherapy for esophageal cancer. When bone marrow suppression occurs, hematopoietic stem cells cannot produce adequate number of blood cells who have normal function, resulting in complications such as anemia, infection, and bleeding; these complications lower the survival of the patient severely. Several studies show that inflammation factors in the blood (for example, NLR, lymphocyte count, and neutrophil count) can predict the prognosis of patients with a variety of tumors [28–31]. Lymphocytes are related to host immunity. Lymphopenia has a negative impact on cellular immunity [32]. Increasing evidence shows that lymphopenia during CCRT in cancer patients is related to tumor prognosis and pathological reactions [33–35]. In all kinds of cancers (including EC), treatment-induced lymphopenia has a close connection with adverse outcomes [14, 36–40].

Platelets contribute to inflammation and immunomodulatory processes. It is reported that the platelet count in cancer patients will increases by about 10–57% [41]. Platelets by serving as a barrier to immune escape promote development of tumor, which can lead to abnormal vasculature and release the secreted factors [1, 42, 43].

Our study believes that age and *Δ*PLR are independent influencing factors of OS, and the OS in the low *Δ*PLR group is better than the high one (95% CI: 12.838-17.162, *P* < 0.001); and *Δ*CLC% is an independent influencing factor of DFS; the low *Δ*CLC% group had better DFS (95% CI: 8.340-13.66, *P* < 0.001). The study of Liang et al. is consistent with ours, in ESCC patients receiving radiotherapy or chemoradiation, NLR, ALC before treatment, NLR and *Δ*NLR after treatment are all significant for the short-term survival of patients [44]. Research on limited-stage small-cell lung cancer by Yu et al. also showed that CLC and PLR are related to prognosis, and higher NLR and PLR are related to decreased survival rate [31]. Research included patients with esophageal and junctional adenocarcinoma (OJA) treated with neoadjuvant chemotherapy shows that PLR is related to poor OS and DFS [45].

A 2015 study that includes 86 esophageal cancer patients who have CRT have the same idea with our study. The high PLR and NLR are related to inferior survival [46].

In our study, we also found that *Δ*PLR is related to the pulmonary side effects of patients after CCRT. In the high *Δ*PLR group, there were 25 patients with radiation pneumonitis 2 and above after CCRT, while the low *Δ*PLR group had 11 patients; the high *Δ*PLR patients were more possibly to develop radiation pneumonitis (*P* = 0.027) (Table 4). We temporarily do not found research on the relationship between radiation pneumonitis and PLR. A study by Dong et al. believes that PLT is related to the occurrence of esophageal fistula during CCRT. Patients with PLT > 153 are more likely to develop fistula than those with PLR ≤ 153 (*P* < 0.001); the study included 379 patients with esophageal cancer; analyzed the relationship between NLR, PLR, MLR, and esophageal fistula; and finally found that PLR is an independent predictor of EC patients receiving CCRT [47]. Unfortunately, this study did not find a correlation between other inflammatory indicators and radiation esophagitis. It may be related to the fact that fewer patients were included in this study.

## 5. Conclusion

Our study found that age and *Δ*PLR are independent factors of OS in patients with ESCC treated with CCRT, and *Δ*CLC% is an independent factor of DFS. And we compared the DFS and OS with *Δ*PLR and *Δ*CLC% andfound that lower *Δ*PLR and *Δ*CLC% is associated with a better survival. And T3-4N1-2 patients in the low *Δ*PLR group and low *Δ*CLC% group have greater survival benefit. Nevertheless, these results are preliminary and need to be validated. The large-scale prospective clinical trials are needed to verify the result.

## Figures and Tables

**Table 1:** Clinical baseline data.

Characteristics	Patients (%), *N* = 106
Sex	
Male	102 (96.2)
Female	4 (3.7)
Age (years)	
≥65	32 (30.2)
<65	74 (69.8)
Tumor T stage	
1	2 (1.9)
2	35 (33)
3	35 (33)
4	34 (32.1)
Tumor N stage	
0	14 (13.2)
1	69 (65.1)
2	23 (21.7)
Tumor TNM stage	
1	6 (5.7)
2	54 (50.9)
3	46 (43.4)
Alcohol consumption	
Yes	78 (73.6)
No	28 (26.4)
Smoking	
Yes	89 (84)
No	17 (16)
Treatment	
Concurrent CRT	106 (100)

**Table 2:** Univariate analysis on influencing factors of OS and PFS.

Factor	OS			PFS		
	*B*	Exp(*B*)	*P* value	*B*	Exp(*B*)	*P* value
Sex	-0.428	0.652	0.468	-0.432	0.649	0.467
Age	0.042	0.959	0.019	-0.034	0.966	0.059
Alcohol consumption	0.243	1.274	0.316	0.169	0.492	1.185
Smoking	0.346	1.414	0.265	0	1	0.999
T stage	-0.021	0.979	0.867	0.046	1.047	0.729
N stage	0.107	1.113	0.536	0.035	1.036	0.846
Tumor TNM stage	0.161	1.174	0.369	0.108	1.114	0.558
*Δ*PLR	-0.001	0.999	<0.001	-0.001	0.999	<0.001
*Δ*PLR%	-0.001	0.999	<0.001	-0.001	0.999	<0.001
*Δ*NLR	-0.025	0.975	<0.001	-0.018	0.982	0.004
*Δ*NLR%	-0.038	0.962	0.003	-0.028	0.972	0.029
*Δ*CLC	0.501	1.650	0.002	0.490	1.633	0.002
*Δ*CLC%	0.041	1.042	<0.001	0.039	1.039	<0.001
*Δ*PLT	-0.001	0.999	0.414	0	1	0.886
*Δ*PLT%	-0.003	0.997	0.350	0	1	0.937

**Table 3:** Multivariate analysis on influencing factors of OS and PFS.

Factor	OS			PFS		
	*B*	Exp(*B*)	*P* value	*B*	Exp(*B*)	*P* value
Age	-0.040	0.961	0.028	0.036	1.037	0.408
*Δ*PLR	-0.002	0.998	0.030	-0.001	0.999	0.345
*Δ*PLR%	0.001	1.001	0.393	0	1.000	0.701
*Δ*NLR	0.026	1.027	0.367	0.004	1.004	0.892
*Δ*NLR%	0.000	1.000	0.993	0.021	1.022	0.528
*Δ*CLC	0.063	1.065	0.822	0.059	1.061	0.826
*Δ*CLC%	0.017	1.017	0.364	0.043	1.044	0.024

**Table 4:** Comparison of clinical baseline data between ΔPLR ≥ 290 and ΔPLR < 290.

Factor	ΔPLR ≥ 290 (*n* = 53)	ΔPLR < 290 (*n* = 53)	*P* value
Sex			0.618
Male	50 (94.3%)	52 (98.1%)	
Female	3 (5.7%)	1 (1.9%)	
Age (years)			1
≥61	16 (30.2%)	16 (30.2%)	
<61	37 (69.8%)	37 (69.8%)	
Tumor T stage			0.982
1	1 (1.9%)	1 (1.9%)	
2	18 (34%)	17 (32.1%)	
3	18 (34%)	17 (32.1%)	
4	16 (30.1%)	18 (34%)	
Tumor N stage			0.461
0	5 (9.4%)	9 (17%)	
1	37 (69.8%)	32 (60.4%)	
2	11 (20.8%)	12 (22.6%)	
Tumor TNM stage			0.923
1	3 (5.7%)	3 (5.7%)	
2	26 (49.1%)	28 (52.8%)	
3	24 (45.3%)	22 (41.5%)	
Alcohol consumption			1
Yes	39 (73.6%)	39 (73.6%)	
No	14 (26.4%)	14 (26.4%)	
Smoking			0.791
Yes	45 (84.9%)	44 (83%)	
No	8 (15.1%)	9 (17%)	
Pre-CLC	1.697 ± 0.688	1.854 ± 0.654	0.228
Pre-PLT	264 ± 90.731	237 ± 74.592	0.109
Radiation pneumonia			*0.027*
0–1	28	42	
2–3	25	11	
Radiation esophagitis			0.407
1	27	30	
2	15	18	
3	8	3	
4	3	2	

**Table 5:** Comparison of clinical baseline data between ΔCLC% ≥ 75 and ΔCLC% < 75.

Factor	ΔCLC% ≥ 75 (*n* = 52)	ΔCLC% < 75 (*n* = 54)	*P* value
Sex			0.672
Male	51 (96.2%)	51 (96.2%)	
Female	2 (3.8%)	2 (3.8%)	
Age			0.768
≥65	15 (28.3%)	17 (32.1%)	
<65	37 (71.7%)	37 (67.9%)	
Tumor T stage			0.420
1	2 (3.8%)	0 (0%)	
2	15 (28.8%)	20 (37%)	
3	17 (32.7%)	18 (33.3%)	
4	18 (34.6%)	16 (29.6%)	
Tumor N stage			0.258
0	4 (7.7%)	9 (18.5%)	
1	36 (69.2%)	32 (61.1%)	
2	12 (23.1%)	12 (20.4%)	
Tumor TNM stage			0.674
1	2 (3.8%)	4 (7.4%)	
2	26 (50%)	28 (51.9%)	
3	24 (46.2%)	22 (40.7%)	
Alcohol consumption			0.768
Yes	38 (73.6%)	40 (73.6%)	
No	14 (26.4%)	14 (26.4%)	
Smoking			0.763
Yes	44 (84.9%)	45 (83%)	
No	8 (15.1%)	9 (17%)	
Radiation pneumonia			0.370
0	1	0	
1	30	39	
2	18	13	
3	3	2	
Radiation esophagitis			0.315
1	24	33	
2	18	15	
3	6	5	
4	4	1	

**Table 6:** Subgroup analysis of *Δ*PLR.

Factor	OS				DFS			
	ΔPLR ≥ 290 (*n* = 53)	ΔPLR < 290 (*n* = 53)	*χ* ^2^	*P* value	ΔPLR ≥ 290 (*n* = 53)	ΔPLR < 290 (*n* = 53)	*χ* ^2^	*P* value
Age								
≥61	27	27	23.3	<0.001	27	27	21.244	<0.001
<61	26	26	25.637	<0.001	26	26	8.895	0.003
Tumor T stage								
1–2	19	18	9.001	0.003	19	18		0.091
3	18	17	24.484	<0.001	18	17	29.506	<0.001
4	16	18	11.893	0.001	16	18	5.858	0.016
Tumor N stage								
0	5	9	1.847	0.174	5	9	0.318	0.573
1	37	31	27.019	<0.001	37	31	22.935	<0.001
2	11	12	7.111	0.008	11	12	5.209	0.022

**Table 7:** Subgroup analysis of *Δ*CLC%.

Factor	OS				DFS			
	ΔCLC% ≥ 74 (*n* = 52)	ΔCLC% < 74 (*n* = 54)	*χ* ^2^	*P* value	ΔCLC% ≥ 74 (*n* = 52)	ΔCLC% < 74 (*n* = 54)	*χ* ^2^	*P* value
Age (years)				0.768				
>61	22	27	20.011	<0.001	22	27	20.113	<0.001
≤61	30	27	15.627	<0.001	30	27	20.113	<0.001
Tumor T stage								
1–2	17	20	6.601	0.01	17	20	4.146	0.042
3	17	18	17.459	<0.001	17	18	18.665	<0.001
4	18	16	17.018	<0.001	18	16	16.230	<0.001
Tumor N stage								
0	4	10	6.314	0.012	4	10	3.543	0.06
1	36	33	5.105	<0.001	36	33	22.960	<0.001
2	12	11	25.437	0.024	12	11	7.488	0.006

## Data Availability

The data used to support the findings of this study are included within the article.
